# Seroprevalence and Placental Transfer of Zika and Dengue Virus Antibodies in Postpartum Women in Southeast Brazil

**DOI:** 10.1002/jmv.70384

**Published:** 2025-05-21

**Authors:** Rafael Rahal Guaragna Machado, Marcella Sanches Peres, Samuel Santos Pereira, Ralyria Mello, Danielle Bastos Araujo, Camila Soares Pereira, Boris Pastorino, Danielle Bruna Leal Oliveira, Michele da Silva Jordan Faleiros, Rossana Pulcineli Vieira Franscisco, Carlos Tadashi Yoshizaki, Silvia Maria Ibidi, Xavier de Lamballerie, Luís Carlos de Sousa Ferreira, Edison Luiz Durigon

**Affiliations:** ^1^ Department of Microbiology, Institute of Biomedical Sciences University of São Paulo São Paulo Brazil; ^2^ Unité des Virus Émergents (UVE: Aix‐Marseille Univ, Università di Corsica, IRD 190, Inserm 1207, IRBA) Marseille France; ^3^ Hospital Israelita Albert Einstein São Paulo São Paulo Brazil; ^4^ Division of Pediatrics, Hospital Universitário University of São Paulo São Paulo Brazil; ^5^ Departamento de Obstetrícia e Ginecologia Disciplina de Obstetrícia, Faculdade de Medicina da Universidade de São Paulo São Paulo Brazil; ^6^ Division of Obstetrics and Gynaecology, Hospital Universitário University of São Paulo São Paulo Brazil; ^7^ Institut Pasteur de São Paulo São Paulo Brazil

**Keywords:** antibodies, dengue virus, seroprevalence, transplacental transfer, Zika virus

## Abstract

Zika (ZIKV) and dengue (DENV) viruses are major public health concerns in Brazil. Understanding population serological status, particularly in pregnant women, is vital for estimating their spread. This study assessed ZIKV and DENV seroprevalence in pregnant women and the transplacental transfer of antibodies to their newborns, crucial for early‐life protection. A cross‐sectional seroprevalence study was conducted with 601 postpartum women and their newborns in São Paulo city, Brazil post‐ZIKV fever outbreak. Paired maternal and umbilical blood samples were collected for ZIKV and DENV antibody testing, which was conducted using ELISA and virus neutralization tests. Maternal and neonatal sociodemographic and clinical data were obtained from interviews and medical records. ZIKV and DENV neutralizing antibodies (nAbs) were detected in 2.4% and 31.6% of participants, respectively. Maternal place of birth, parity, education level, and prenatal Toxoplasmosis serology were identified as risk factors associated with ZIKV and/or DENV infection. Effective transplacental transfer of specific ZIKV and DENV antibodies from mothers to newborns was observed. Nulliparous women and those with a history of DENV infection exhibited higher transfer ratios (TR) of DENV antibodies. Low‐birthweight and preterm neonates had lower DENV‐1 antibody TRs than heavier and term infants. Low seroprevalence of ZIKV and DENV antibodies in the study population indicates a high vulnerability to infection by these viruses. Maternal and neonatal characteristics were associated with seropositivity for ZIKV and DENV and the efficiency of DENV antibody transfer to neonates. These findings can guide public health strategies for evaluating the TR effectiveness of antibodies following future ZIKV and DENV vaccination programs in pregnant women.

## 1 Introduction

Zika virus (ZIKV) and dengue virus (DENV), both members of the family *Flaviviridae*, genus *Orthoflavivirus*, represent significant public health concerns, particularly in tropical and subtropical regions such as Brazil [1]. Both arboviruses are transmitted by *Aedes* mosquitoes [2] and are responsible for severe outbreaks, often placing a substantial burden on public health systems. ZIKV infection during pregnancy has been linked to congenital Zika syndrome (CZS), which is characterized by microcephaly and other neurological defects [3]. Similarly, DENV infections can result in life‐threatening conditions such as severe dengue or dengue hemorrhagic fever [4].

The co‐circulation of these viruses in endemic areas, such as São Paulo, the most populous city in southeastern Brazil, presents unique challenges for public health management and maternal‐fetal health. The neonate's passive immunity, acquired primarily by transplacental transfer of maternal immunoglobulin G (IgG) antibodies [5], offers temporary protection against infections during the first few months of life [6]. However, the efficiency of placental transfer can be influenced by multiple factors, including gestational age, IgG subclass, antigen specificity, maternal antibody titers, and placental health [7].

A comprehensive understanding of the antibody transfer dynamics in the context of ZIKV and DENV infection is of paramount importance, as it can potentially inform vaccine development strategies in endemic regions and guide the development of more effective neonatal infection prevention measures. Despite the importance of maternal and neonatal immunity to these arboviruses, only few studies have focused on the seroprevalence of ZIKV and DENV infection in postpartum women and the efficiency of antibody transfer to their newborns. Moreover, factors that may impact the placental transfer of ZIKV and DENV‐specific antibodies, such as maternal antibody levels, chronic infections, and placental pathology, remain underexplored.

This study aims to investigate the seroprevalence of ZIKV and DENV antibodies in postpartum women in São Paulo city and assess the placental transfer of these antibodies to their newborns. By evaluating the factors influencing transplacental antibody transfer, we hope to contribute to a better understanding of maternal and neonatal immunity in the context of ZIKV and DENV exposure, with implications for vaccine design and public health strategies in regions where both viruses circulate.

## 2 Methods

### 2.1 Study Design and Population

A cross‐sectional seroprevalence study was conducted in São Paulo city, in the Southeast region of Brazil (Figure S1), between December 2018 and January 2020. The study was conducted at University Hospital of the University of São Paulo (HU/USP), an academic public hospital with an average of 2500 deliveries annually and a center of excellence for maternal‐neonatal health research. Parturient women admitted for delivery at HU/USP were invited to participate in the study. A simple random sampling technique was employed, allowing participation across all three daily shifts and on any day of the week. Twelve cases were excluded due to the absence of maternal serum or cord blood samples (Figure S2). Following the obtaining of informed consent, maternal and cord blood samples were collected during routine screening tests to measure ZIKV and DENV‐specific antibodies. To ensure the confidentiality of the participants, all samples were fully deidentified before antibody analysis. A structured questionnaire was administered within 48 h of delivery to gather clinical and demographic information, which was cross‐verified with data from patient‐held antenatal clinic records and hospital charts.

### 2.2 Sample Size Calculation

The sample size of the population was calculated based on an expected seroprevalence of 50%, an absolute error of 4%, and a 95% confidence level, resulting in a required sample size of 601 participants. It is important to note that, due to the lack of data in the literature regarding the seroprevalence of ZIKV and DENV in pregnant women in the study region at the time of the recruitment, the maximum variability (50% prevalence) was adopted.

### 2.3 Sample Collection

To minimize the invasiveness of sample collection, venous blood samples were obtained from all parturients as part of the standard protocol at HU‐USP for serological testing of HIV and *Treponema pallidum*. Umbilical cord blood from the newborn was collected by clamping the cord, cutting it, and draining the blood into sterile collection tubes. The blood samples were placed in EDTA tubes and stored at room temperature for up to 6 h before being centrifuged at 1500*g* for 10 min at RT to separate the serum. The serum samples were then stored at −80°C until further use.

### 2.4 Antibody Measurement

Before any testing, all samples were heat‐inactivated (decomplemented) at 56°C for 30 min and subsequently stored at −80°C. For the detection of anti‐ZIKV and anti‐DENV IgM antibodies, the anti‐Zika virus IgM μ‐capture ELISA kit (#ab213327, Abcam, Cambridge, USA) and the Panbio Dengue IgM Capture ELISA kit (#01PE20, Abbott, Chicago, IL, USA) were used, respectively, following the manufacturer's instructions. The detection of anti‐ZIKV IgG antibodies was conducted using a NS1‐based commercial ELISA, the Zika‐v IgG kit (#ZKE‐096, Advagen Biotech Ltda, Itu, Brazil), in accordance with the instructions provided by the manufacturer. Furthermore, an EDIII‐based in‐house ELISA assay was conducted for the detection of anti‐DENV 1‐4 IgG antibodies, as previously described and validated [8].

ZIKV and DENV 1‐4 nAbs titers were measured using the cytopathic viral neutralization test (CPE‐VNT), as previously described [9]. Briefly, 96‐well plates were seeded with Vero cells (ATCC CCL‐81) and incubated until reaching 80%–90% confluence. Heat‐inactivated serum samples were serially diluted (1:40 to 1:1280) and mixed with ZIKV or DENV 1–4 at a concentration of 1 × 10^3^ TCID50/mL to achieve 50–100 viral particles per well. After 1 h of incubation, 150 µL of the serum‐virus mixture was transferred to the cell plates and incubated for 4 days for ZIKV, and 4 to 7 days for DENV 1–4. The virus neutralization titer (VNT_100_) was determined as the highest serum dilution that achieved 100% viral neutralization (absence of CPE). Serum samples with a VNT100 ≥ 40 were classified as positive [9]. A second test, the plaque reduction neutralization test (PRNT) was conducted on samples exhibiting discordant results between the ELISA (IgG) and the CPE‐VNT (nAbs), as previously described [10]. Vero cells (ATCC CCL‐81) were seeded in 24‐well plates at a density of 1 × 10^5^ cells/well and incubated at 37°C with 5% CO₂ for 24–48 h. Heat‐inactivated serum samples were serially diluted six times (from 1:20 to 1:6,280). Virus stocks at 600 PFU/mL were added to the diluted sera to reach 20–40 PFU/well, and the mixture was incubated at 37°C with 5% CO₂ for 1 h to allow neutralization. Then, 100 µL of the serum‐virus mixture (in duplicate) was added to the Vero cell monolayer and incubated for 1 h at 37°C with 5% CO₂ for adsorption. After that, 1 mL/well of 2% CMC was added. Plates were incubated at 37°C with 5% CO₂ for 4 days for ZIKV, and 4–7 days for DENV 1–4. Finally, the plates were washed with PBS (pH 7.2), fixed, and stained with Naphtol Blue Black (Sigma‐Aldrich). The PFUs were counted, and the nAb titers were calculated. The PRNT results were interpreted following WHO criteria, which define a recent infection as a PRNT_90_ titer≥ 20, indicating a 90% reduction in plaques compared to the control. The same viral strains were employed for both tests, namely ZIKV (MH882527.1), DENV‐1 (AB074760.1), DENV‐2 (KM204118.1), DENV‐3 (KC425219.1), and DENV‐4 (GU289913.1).

### 2.5 Statistical Analyses

Categorical variables were summarized as counts (*n*) and percentages (%), with group comparisons using Chi‐square or Fisher's Exact tests, as appropriate. Continuous variables were expressed as means ± standard deviation (for normal distributions) or medians with interquartile range (for non‐normal distributions), with *t*‐tests for normal and Wilcoxon rank‐sum tests for nonparametric data. Multivariable models were used to predict maternal seroprevalence and identify factors associated with placental transfer, including variables with *p* < 0.2 from univariate analysis. Antibody titers were log2‐transformed, and geometric mean titers (GMTs) with 95% confidence intervals (CIs) were calculated. Placental transfer efficiency (TR) was calculated as the percentage ratio of umbilical cord to maternal antibody concentrations. Statistical significance was set at *p* < 0.05, with results reported alongside 95% CIs. Analyses and graphs were generated using GraphPad Prism 8.2.

## 3 Results

### 3.1 Cohort Characteristics

Between December 2018 and January 2020 (14 months), a total of 601 postpartum women and their respective newborns (*n* = 609) were enrolled in the study. Maternal sociodemographic characteristics are shown in Table 1. The prevalence of positive maternal ZIKV and DENV IgG was higher in women originally from northeast region of Brazil (OR 12.79 and 3.05, respectively) compared to women from South/Southeast region (Table 1). ZIKV and DENV seropositive participants were more likely to be living with their partners (OR 7.36 and 1.54, *p *= 0.02) compared to women living separately or alone. Women with lower education (less than 9 years) had 1.8 times (95% CI, 1.1–2.8, *p *= 0.01) greater odds of being DENV seropositive than participants with higher education level (Table 1). These associations remained independently significant in the multivariable logistic regression model (Table S1).

**Table 1:** Sociodemographic characteristics of the participants (*n* = 601). Comparison between postpartum women with and without anti‐ZIKV or anti‐DENV nAbs detected.

Maternal characteristic	No (%)	ZIKV				DENV			
	Total (*n* = 601)	Seropositive (*n* = 17)	Seronegative (*n* = 584)	OR (95% CI)	*p* value*	Seropositive (*n* = 190)	Seronegative (*n* = 411)	OR (95% CI)	*p* value*
Age (in years), median (IQR)	26 (22–31)	24 (22.5–26.5)	27 (22–31)	—	0.17**	26 (22‐31)	27 (22‐32)	—	0.38**
14–24	248 (41.2)	9 (52.9)	239 (40.9)	Ref	—	82 (43.2)	166 (40.4)	Ref	—
25–35	281 (46.8)	6 (35.3)	275 (47,1)	0.58 (0.20–1.55)	0.43	91 (47.9)	190 (46.2)	0.97 (0.67–1.40)	0.92
> 35	72 (12.0)	2 (11.8)	70 (12.0)	0.76 (0.16–2.99)	> 0.99	17 (8.9)	55 (13.4)	0.62 (0.35–1.15)	0.14
Ethnicity									
White	412 (68.5)	13 (76.5)	399 (41.1)	Ref	—	128 (67.4)	284 (69.1)	Ref	—
Black	21 (3.5)	1 (5.9)	20 (8.3)	1.53 (0.14–9.24)	0.50	4 (2.1)	17 (4.1)	0.52 (0.19–1.57)	0.33
Asian or Mixed	168 (27.9)	3 (17.6)	165 (50.6)	0.56 (0.17–1.93)	0.57	58 (30.5)	110 (26.7)	1.17 (0.79–1.72)	0.43
Place of birth									
Other country	10 (1.7)	1 (5.9)	9 (1.5)	Ref	—	1 (0.5)	9 (2.2)	Ref	—
Brazil	591 (98.3)	16 (94.1)	575 (98.5)	0.25 (0.03–2.91)	0.25	189 (99.5)	402 (97.8)	4.23 (0.70–46.70)	0.18
*South or Southeast region*	425 (71.9)	3 (18.7)	422 (73.4)	Ref	—	105 (55.5)	320 (79.6)	Ref	—
*Northeast region*	156 (26.4)	13 (81.3)	143 (24.9)	**12.79 (3.69–42.48)**	**< 0.01**	78 (41.3)	78 (19.4)	**3.05 (2.08–4.48)**	**< 0.01**
*North or Midwest region*	10 (1.7)	0 (0.0)	10 (1.7)	—	—	6 (3.2)	4 (1.0)	**4.57 (1.20–14.52)**	**0.02**
Civil status									
Single	485 (80.7)	12 (70.6)	473 (81.0)	Ref	—	154 (81.1)	330 (80.3)	Ref	—
Married	116 (19.3)	5 (29.4)	111 (19.0)	1.77 (0.68–5.01)	0.34	35 (18.4)	81 (19.7)	0.93 (0.60–1.43)	0.82
Marital status									
Not living together	185 (30.8)	1 (5.9)	184 (31.5)	Ref	—	47 (24.7)	138 (33.6)	Ref	—
Living together (consensual union)	416 (69.2)	16 (94.1)	400 (68.5)	**7.36 (1.26–78.00)**	**0.02**	143 (75.3)	273 (66.4)	**1.54 (1.04–2.28)**	**0.02**
Education level, years									
≥ 12 (CHS, IC and/or CC)	333 (55.4)	12 (70.6)	321 (55.0)	Ref	—	92 (48.4)	241 (58.6)	Ref	—
≥ 9 e < 12 (CMS and/or IHS)	161 (26.8)	4 (23.5)	157 (26.9)	0.68 (0.24–2.12)	0.59	55 (28.9)	106 (25.8)	1.36 (0.90–1.11)	0.14
< 9 (NS, IES, CES and/or IMS)	107 (17.8)	1 (5.9)	106 (18.1)	0.25 (0.02–1.64)	0.20	43 (22.6)	64 (15.6)	**1.76 (1.13–2.77)**	**0.01**
Professional occupancy									
Housewife	198 (33.0)	4 (23.5)	194 (33.2)	Ref	—	64 (33.7)	134 (32.6)	Ref	—
Work outside the home	300 (49.9)	12 (70.6)	288 (49.3)	2.02 (0.65–5.80)	0.30	99 (52.1)	201 (48.9)	1.03 (0.71–1.52)	0.92
Does not work (unemployed or student)	103 (17.1)	1 (5.9)	102 (17.5)	0.47 (0.04–2.92)	0.66	27 (14.2)	76 (18.5)	0.74 (0.43–1.26)	0.29

Abbreviations: CC, complete college; CES, complete elementary school; CHS, completed high school; CI, confidence interval; CMS, complete middle school; DENV, dengue virus; IC, incomplete college; IHS, incomplete high school; IMS, incomplete middle school; IPS, incomplete elementary school; IQR, interquartile range; nAbs, neutralizing antibodies; NS, no schooling; OR, odds ratio; Ref, reference; ZIKV, Zika virus.

* The *p* values were calculated using Fisher's exact test (two‐sided method). OR Confidence intervals (CIs) were computed using the Baptista‐Pike method.

** The *p* value was determined by the nonparametric Mann–Whitney test (continuous variable).

Regarding the clinical characteristics of the mothers, primiparous (first‐time mother) and nulliparous (never been pregnant or never given birth to a viable child) women had 4.0 times (95% CI, 1.39–11.51; *p* = 0.01) and 3.4 times (95% CI, 1.19–9.89; *p* = 0.02), respectively, greater odds of being ZIKV seropositive compared to multiparous participants (Table S2). However, these associations did not remain independently significant in the multivariable logistic regression model (Table S3). Regarding symptoms of arboviral diseases and risk factors for infection during pregnancy, parturient who reported traveling during pregnancy had lower odds of DENV seropositivity compared to those who did not travel (OR 0.6, 95% CI: 0.4–0.9, *p* = 0.01; Table S3). Additionally, a personal history of ZIKV infection (*p *= 0.0283) was positively associated with ZIKV seropositivity, as was a previous DENV infection with DENV seropositivity (*p *< 0.0001; Table S3). No other variables analyzed were significantly associated with the serological profile for ZIKV or DENV (*p *> 0.05; Table S3).

None of the participants tested positive for HIV serology (Table S4), and thus this variable was not analyzed. Additionally, among participants with available prenatal serological data, antibodies for syphilis, hepatitis B, toxoplasmosis, and group B streptococci (GBS) did not demonstrate a significant correlation with ZIKV seropositivity (*p *> 0.05, Table S4). However, women with toxoplasmosis showed a trend toward a higher likelihood of DENV positivity (OR 1.49, *p* = 0.04, Table S4). This association remained independently significant in the multivariable logistic regression model (Table S4).

The study included 609 neonates, comprising 594 singletons, 6 twin pairs, and 1 triplet set. The anthropometric parameters and general clinical characteristics of the newborns cohort are summarized in Table 2. As for the newborns' anthropometric characteristics, none showed no significant correlations were found with the serological status of their mothers for ZIKV or DENV, including the occurrence of microcephaly (*p *> 0.05; Table 2). However, among neonatal clinical features, dermatological alterations were positively associated with maternal DENV seropositivity (Table 2). Newborns presenting dermatological changes had 1.7 times higher odds of being DENV‐seropositive compared to those without such changes (95% CI, 1.2–2.4; *p* < 0.01; Table 2). This association remained statistically significant in the multivariable logistic regression analysis, with an adjusted odds ratio of 1.64 (95% CI, 1.15–2.34; *p* = 0.006; Table S5). All other clinical characteristics showed no significant associations with maternal serological status for the studied arboviruses (*p *> 0.05; Table 2).

**Table 2:** Anthropometric and clinical characteristics of the 609 newborns participating in the study potentially correlated with maternal seropositivity to ZIKV (*n* = 17) and DENV (*n* = 193). Anthropometric characteristics were represented by mean standard deviation (SD) and 95% confidence interval (95% CI) while the clinical characteristics by sample number (*n*) and percentage (%).

Neonatal characteristic	No (%) or mean ± SD (95% CI)	ZIKV				DENV			
	Total (*n* = 609)	Seropositive (*n* = 17)	Seronegative (*n* = 592)	OR (95% CI)	*p* value*	Seropositive (*n* = 193)	Seronegative (*n* = 416)	OR (95% CI)	*p* value*
Weight (kg)	3.32 ± 0.47 (3.28–3.35)	3.28 ± 0.45 (3.05–3.51)	3.32 ± 0.47 (3.28–3.36)	—	0.54	3.29 ± 0.48 (3.23–3.37)	3.32 ± 0.46 (3.28–3.37)	—	0.58
Length (cm)	49.0 ± 2.2 (48.8–49.2)	48.3 ± 3.4 (46.5–50.1)	49.0 ± 2.2 (48.8–49.2)	—	0.51	48.9 ± 2.1 (48.7–49.3)	49.0 ± 2.3 (48.8–49.2)	—	0.55
Head circumference (cm)	34.2 ± 1.3 (34.1–34.3)	34.0 ± 1.3 (33.3–34.6)	34.2 ± 1.3 (34.1–34.3)	—	0.34	34.2 ± 1.4 (34.1–34.4)	34.2 ± 1.3 (34.1–34.3)	—	0.76
Gestational age (wk)	39.1 ± 1.2 (39.0–39.2)	39.1 ± 1.3 (39.0–39.2)	39.1 ± 1.3 (39.0–39.2)	—	0.49	39.1 ± 1.3 (38.9–39.2)	39.2 ± 1.2 (39.1–39.3)	—	0.36
Microcephaly									
No	596 (97.9)	17 (100.0)	579 (97.8)	Ref	—	191 (99.0)	405 (97.3)	Ref	—
Yes	13 (2.1)	0 (0.0)	13 (2.2)	0.00 (0.00–9.3)	> 0.99	2 (1.0)	11 (2.7)	2.59 (0.63–11.82)	0.24
Sex									
Male	290 (47.6)	7 (41.2)	283 (47.8)	Ref	—	89 (41.1)	201 (48.3)	Ref	—
Female	319 (52.4)	10 (58.8)	309 (52.2)	0.76 (0.29–2.03)	0.59	104 (53.9)	215 (51.7)	0.91 (0.65–1.29)	0.61
Apgar indices (1 min)									
0–3	10 (1.6)	0 (0.0)	10 (1.7)	Ref	—	4 (2.1)	6 (1.5)	Ref	—
4–6	16 (2.6)	0 (0.0)	16 (2.7)	1.57 (0.03–85.42)	0.82	2 (1.0)	14 (3.4)	4.67 (0.66–32.74)	0.12
7‐10	583 (95.7)	17 (100.0)	566 (95.6)	1.54 (0.09–27.37)	0.77	187 (96.9)	388 (95.1)	1.38 (0.38–4.96)	0.62
GA × birthweight									
SGA	48 (7.9)	3 (17.6)	51 (8.6)	Ref	—	17 (8.8)	37 (8.9)	Ref	—
AGA	487 (80.0)	14 (82.4)	512 (86.5)	2.15 (0.60–7.73)	0.24	167 (86.5)	359 (86.3)	0.99 (0.54–1.80)	0.98
LGA	74 (12.1)	0 (0.0)	29 (4.9)	4.01 (0.20–80.34)	0.36	9 (4.7)	20 (4.8)	1.02 (0.38–2.70)	0.97
Neonatal RDS									
No	509 (83.6)	16 (94.1)	493 (82.4)	Ref	—	165 (85.5)	344 (82.7)	Ref	—
Yes	100 (16.4)	1 (5.9)	99 (17.6)	0.31 (0.04–2.37)	0.26	28 (14.5)	72 (17.3)	0.81 (0.50–1.30)	0.38
Dermatological changes									
No	275 (45.2)	7 (41.2)	274 (45.5)	Ref	—	70 (36.3)	206 (49.5)	Ref	—
Yes	334 (54.8)	10 (58.8)	318 (54.5)	1.23 (0.46–3.28)	0.68	123 (63.7)	210 (50.5)	**1.72 (1.22–2.43)**	**< 0.01**
Hip dysplasia (Ortolani test)									
Negative	593 (97.4)	17 (100.0)	576 (97.3)	Ref	—	192 (95.5)	401 (96.4)	Ref	—
Positive	16 (2.6)	0 (0.0)	16 (2.7)	0.00 (0.00–7.25)	> 0.99	1 (0.5)	15 (3.6)	0.14 (0.02–1.06)	0.05
Neonatal screening									
Pulse oximetry									
*SpO*_*2*_ < *95%*	8 (1.3)	0 (0.0)	8 (1.4)	Ref	—	2 (10)	6 (1.4)	Ref	—
*SpO*_*2*_ *≥ 95%*	601 (98.7)	17 (100.0)	584 (98.6)	1.96 (0.11–35.41)	0.65	191 (990)	410 (98.6)	0.71 (0.14–3.58)	0.68
OAE testing**									
*Absent OAEs*	13 (2.1)	1 (5.9)	12 (2.1)	Ref	—	3 (1.6)	10 (2.5)	Ref	—
*Present OAEs*	581 (95.4)	16 (94.1)	565 (97.9)	2.94 (0.36–24.02)	0.31	183 (98.4)	398 (97.5)	0.65 (0.18–2.40)	0.52
Red reflex test (RRT)									
*Normal*	605 (99.3)	17 (100.0)	588 (99.3)	Ref	—	3 (1.5)	1 (0.2)	Ref	—
*Abnormal*	4 (0.7)	0 (0.0)	4 (0.7)	0.27 (0.01–5.16)	0.38	190 (98.5)	411 (99.8)	6.49 (0.67–62.80)	0.11

Abbreviations: AGA, adequate for the gestational age; CI, confidence interval; cm, centimeters; DENV Dengue virus; GA, gestational age; kg, kilograms; LGA, large for the gestational age; OAE, otoacoustic emissions; OR, odds ratio; RDS, respiratory distress syndrome; Ref, reference; SGA, small for the gestational age; SpO_2_, peripheral oxyhemoglobin saturation; wk, weeks; ZIKV Zika virus; %, percentage.

* *p* values were determined by the nonparametric Mann–Whitney test for continuous variables, otherwise were calculated using Fisher's exact test (two‐sided method).

** Lack of information in the OAE testing of 15 newborns.

### 3.2 Serological Investigation of ZIKV and DENV Infections

**Figure 1:**
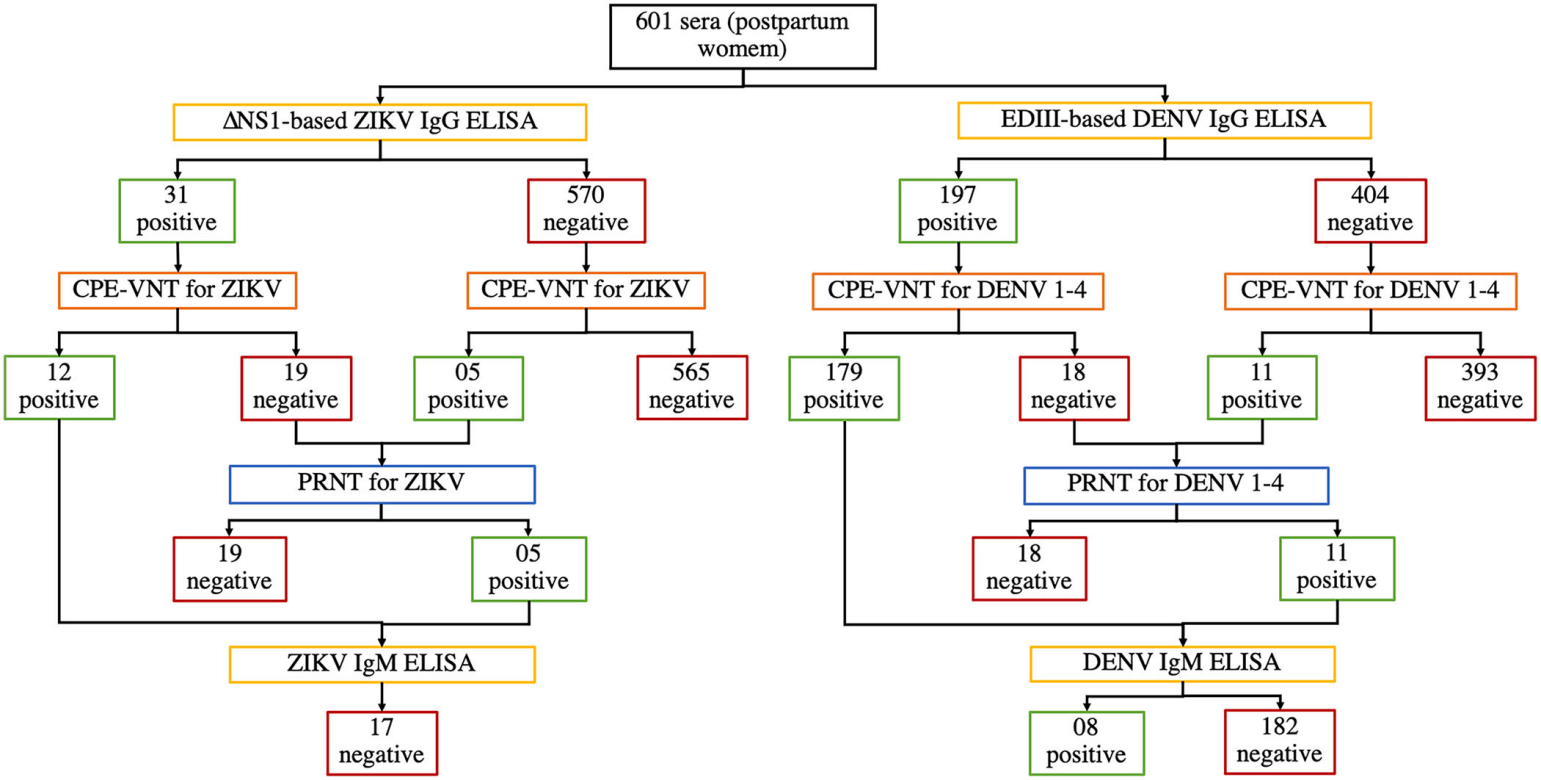
Flowchart of the serological tests conducted to detect recent or past ZIKV and DENV infections in serum samples of all postpartum women participants (*n* = 601). The samples were screened using an ELISA to detect specific IgG antibodies with all positive results confirmed by the CPE‐VNT technique to detect neutralizing antibodies. Discordant samples were further analyzed using the PRNT assay. Once true positive samples were identified they were subsequently tested for the presence of anti‐ZIKV and anti‐DENV specific IgM antibodies.

The serological status for ZIKV and DENV infection was assessed in all the participants (*n* = 601) included in the study (Figure 1). A prevalence of 2.8% (95% CI: 1.8%–4.5%) and 31.61% (95% CI: 28.02%–35.44%), for ZIKV and DENV‐nAbs respectively, was observed. All serum samples were first analyzed using an ELISA to detect anti‐∆NS1 ZIKV and anti‐EDIII DENV IgG antibodies. The samples were then tested using a cytopathic effect‐based virus neutralization test (CPE‐VNT) to confirm ELISA findings (Figure 1). The performance metrics for ELISA are detailed in Table S6 and Figure S3. All samples with discordant results between ELISA and CPE‐VNT were further analyzed using the plaque reduction neutralization test (PRNT) showing 100% concordance with CPE‐VNT results (Figure 1). Additionally, all samples that showed neutralizing antibodies (nAbs) against ZIKV or at least one DENV serotype (VNT_100_ ≥ 40) were tested for IgM antibodies (Figure 1). Anti‐ZIKV IgM antibodies were not detected in all 17 positive samples indicating no evidence of recent infection by ZIKV while in 4.2% (95% CI: 2.02%–5.44%) of the samples with nAbs DENV‐specific IgM antibodies were detected.

It is noteworthy that among the positive ELISA samples for ZIKV that were not confirmed by a neutralization method (n = 19, Figure 1), 84.2% (*n* = 16) tested positive for DENV (anti‐EDIII IgG and DENV‐nAbs). This finding underscores the high level of cross‐reactivity between these arboviruses in serological assays based on the detection of IgG‐binding antibodies. Moreover, all true ZIKV‐positive samples were tested for DENV and showed VNT_100_ < 40 confirming the ZIKV result and excluding potential cross‐reactivity.

**Figure 2:**
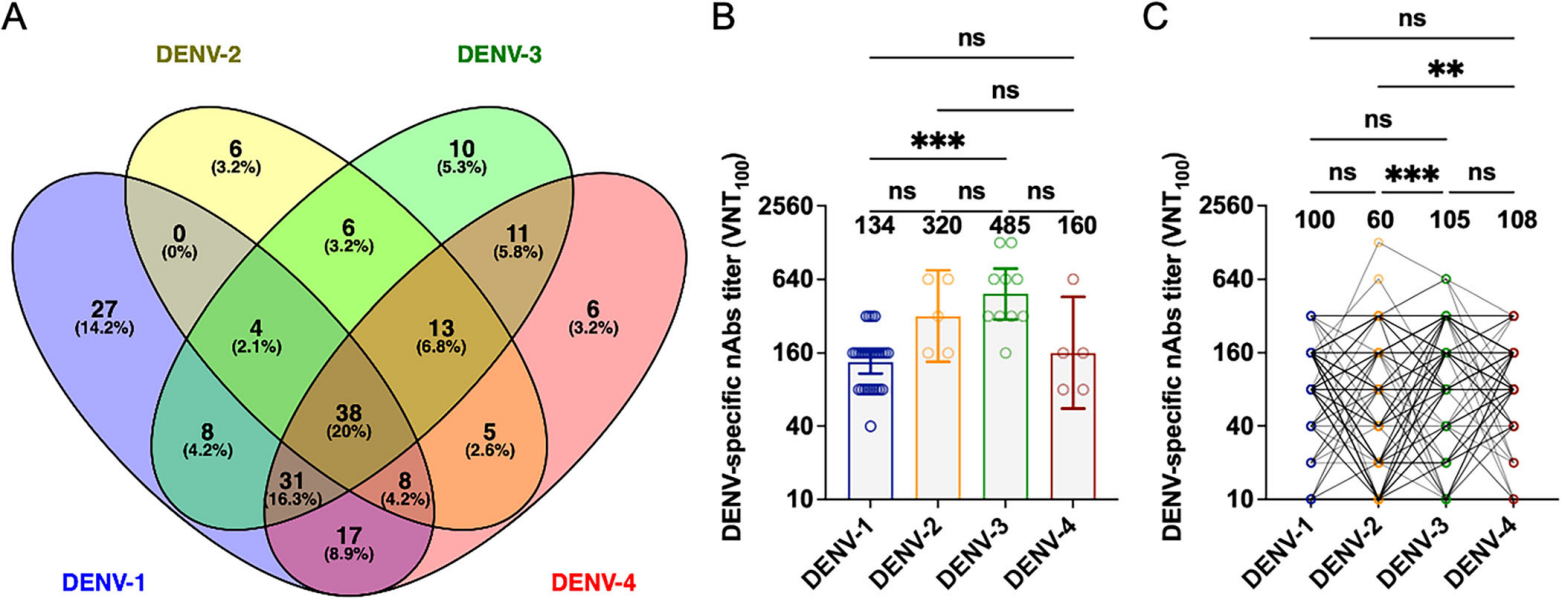
Frequency of monotypic and multitypic DENV infections and comparison of neutralizing antibody (nAb) titers among the four DENV serotypes. (A) Venn diagram illustrating the distribution (absolute number) and percentage (%) of the different DENV serotypes in the study population, based on the following analysis criteria: nAb titers VNT100 ≥ 40 for at least one serotype and/or a ≥ 4‐fold difference in nAb titers. (B) Comparison of nAb titers observed against the different DENV serotypes in monotypic infections (DENV‐1, *n* = 27; DENV‐2, *n* = 06; DENV‐3, *n* = 10, and DENV‐4, *n* = 06). The Friedman test was used for statistical analysis. (C) Comparison of nAb titers observed against the different DENV serotypes in multitypic infections (*n* = 145). (ns, not significant; *p < 0.05; **p < 0.01; ***p < 0.001; ****p < 0.0001). Source: Prepared by the author. The Venn diagram was created using the online Venny software, and graphs (B and C) were generated using GraphPad Prism 8.2 (GraphPad Software, CA, USA).

Among the DENV seropositive samples (*n* = 190), 70%, 42%, 64%, and 68% of patients had nAbs against DENV‐1, DENV‐2, DENV‐3, and DENV‐4, respectively. Regarding the prevalence of monotypic or multitypic responses 74% of patients exhibited a multitypic response while 26% had a monotypic response (Figure 2A). DENV‐1 was the serotype with the highest frequency of monotypic response (14.2%). Among multitypic responses 20% of samples had nAb titers ≥ 40 for all four DENV (Figure 2A). For samples with monotypic responses a significant difference in neutralizing antibody titers was observed between DENV‐1 and DENV‐3 with a higher geometric mean titer (GMT) against DENV‐3 compared to DENV‐1 (Figure 2B). In multitypic responses titers against DENV‐2 were significantly lower than those against DENV‐3 and DENV‐4 (Figure 2C).

### 3.3 Transplacental Transfer of Anti‐ZIKV and Anti‐DENV Antibodies

**Figure 3:**
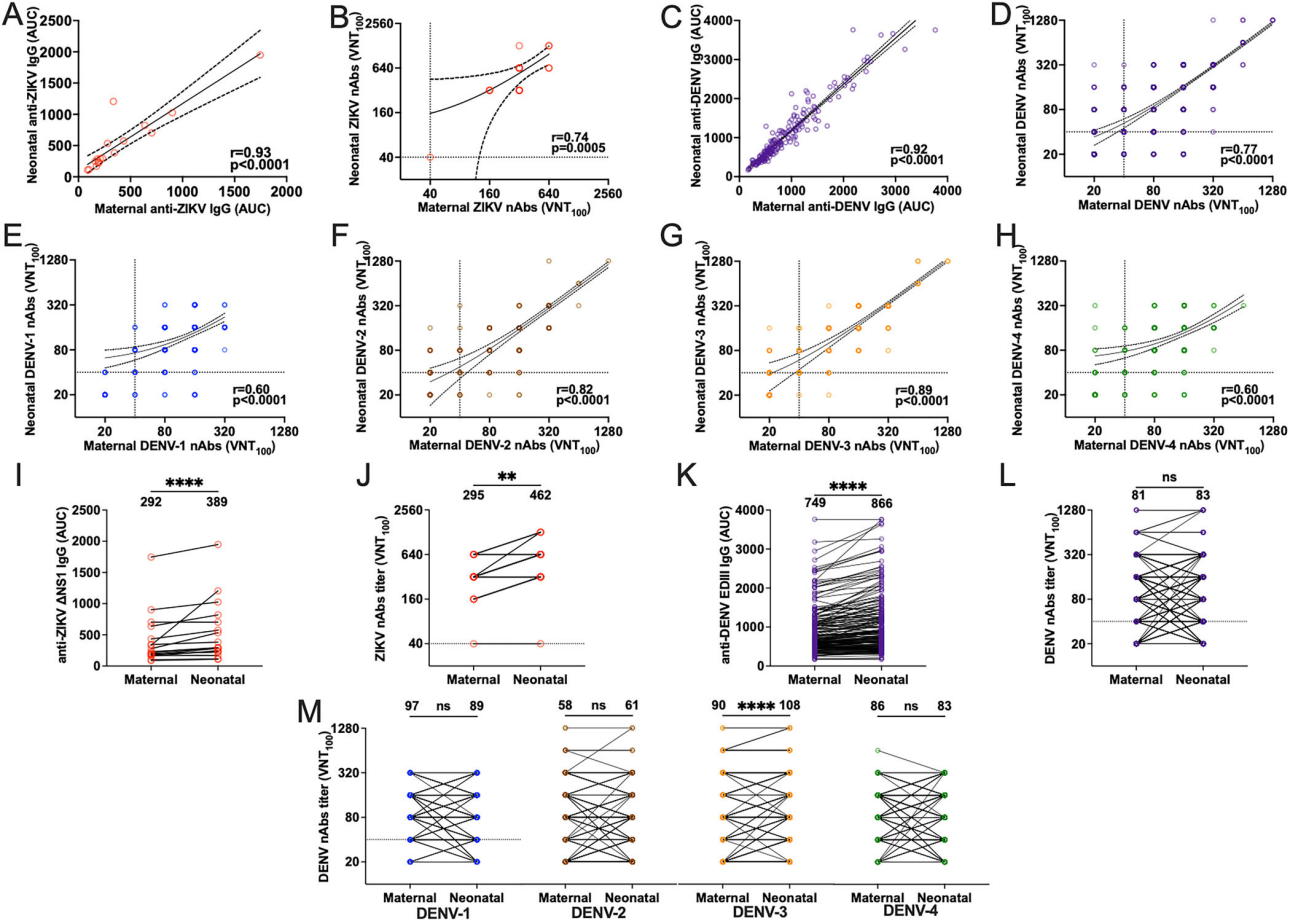
Correlation and comparison of maternal and umbilical cord IgG binding and neutralizing antibody titers in samples positive for the presence of antibodies against ZIKV (*n* = 17) and DENV (*n* = 193). (A‐H) Correlation between the levels of maternal and umbilical cord IgG anti‐NS1 (A and C) and neutralizing (B, D‐H) antibodies in samples positive for antibodies against (A and B) ZIKV (*n* = 17) and (C and D) DENV 1–4 (*n* = 193), (E) DENV‐1 (*n* = 193), (F) DENV‐2 (*n* = 193), (G) DENV‐3 (*n* = 193) and (H) DENV‐4 (*n* = 193). The value of *r* and *p* was calculated using Spearman's correlation. (I–M) Comparison of IgG anti‐NS1 (I and K) and neutralizing (J, L and M) antibody titers between maternal serum samples and umbilical cord samples against (I and J) ZIKV (*n* = 17) and (K and L) DENV 1–4 (*n* = 193), (M) DENV‐1 (*n* = 193), DENV‐2 (*n* = 193), DENV‐3 (*n* = 193), and DENV‐4 (*n* = 193). The *p* value was calculated using the nonparametric Wilcoxon test. ns, not significant; *p < 0.05; **p < 0.01; ***p < 0.001; ****p < 0.0001. *Source:* Prepared by the author using GraphPad Prism 8.2 (GraphPad Software, CA, USA).

Anti‐NS1 ZIKV IgG antibodies and anti‐EDIII DENV‐specific IgG antibodies were detected in all paired maternal‐umbilical cord samples, from ZIKV‐seropositive mothers (*n* = 17) and DENV‐seropositive mothers (*n* = 190), respectively. A strong correlation was observed between maternal and umbilical cord antibody levels for anti‐NS1 ZIKV IgG, *r* = 0.93 (95% CI: 0.80–0.97) and for anti‐EDIII DENV IgG, *r* = 0.96 (95% CI: 0.95–0.97, *p *< 0.0001; Figure 3A,C). The levels of ZIKV and DENV IgG antibodies, measured by the AUC, were significantly higher in the umbilical cord samples (GMT = 389 ± 2.3 and 869 ± 1.9, respectively) compared to the corresponding maternal samples (GMT = 292 ± 2.2 and 753 ± 1.8, respectively; Figure 3I,K), with a transfer ratio (TR) of 133.3% (*p *= 0.001) and 118.6%, respectively (*p *< 0.0001; Table 3).

**Table 3:** Placental transfer rate (TR) of IgG antibodies and neutralizing antibodies (nAbs) specific to ZIKV in 17 paired samples from mothers and umbilical cords.

	Antibodies^a^	Maternal (*n* = 190)	Neonatal (*n* = 193)	TR, % (95% CI)^b^	*p* value*
ZIKV	IgG anti‐NS1	8.2 (7.6–8.8)	8.6 (8.0–9.2)	133.3 (131.0–135.6)	< 0.01
	nAbs	8.2 (7.7–8.7)	8.9 (8.2–9.5)	156.6 (146.5–167.3)	< 0.01
DENV	IgG anti‐EDIII	9.8 (9.7–10.0)	10.9 (9.9–10.2)	118.6 (118.1–119.0)	< 0.01
	nAbs				
	*DENV‐1*	7.1 (7.0–7.2)	7.0 (6.9–7.1)	94.4 (93.0–95.6)	0.24
	*DENV‐2*	7.5 (7.2–7.7)	7.4 (7.1–7.7)	95.5 (91.0–98.6)	0.06
	*DENV‐3*	7.7 (7.5–7.9)	7.9 (7.6–8.1)	113.1 (112.7–113.4)	< 0.01
	*DENV‐4*	7.1 (6.9–7.2)	7.0 (6.9–7.2)	96.6 (95.8–97.3)	0.56

^a^ Antibody titers were transformed into logarithm base 2 (log_2_).

^b^ Transfer rate = (neonatal nAb titer/maternal nAb titer) × 100.

* *p* values were determined by the nonparametric Wilcoxon test (paired samples).

A strong positive correlation were observed between maternal and umbilical cord nAbs levels for ZIKV (*r* = 0.74, 95% CI: 0.39–0.90, *p* = 0.0005; Figure 3B), and for all four DENV serotypes (Figure 3D): DENV‐1 (*r* = 0.60, 95% CI: 0.50–0.69, *p* < 0.0001; Figure 3E), DENV‐2 (*r* = 0.88, 95% CI: 0.84–0.91, *p* < 0.0001; Figure 3F), DENV‐3 (*r* = 0.91, 95% CI: 0.88–0.93, *p* < 0.0001; Figure 3G), and DENV‐4 (*r* = 0.62, 95% CI: 0.53–0.71, *p* < 0.0001; Figure 3H). Similarly, ZIKV nAbs titers were higher in the neonatal samples (GMT = 462 ± 2.0) than in the corresponding maternal samples (GMT = 295 ± 1.9; Figure 3J), with a TR of 156.6% (*p *= 0.002; Table 3). In contrast, between the DENV serotypes (Figure 3L), only for DENV‐3 the nAbs titers were higher in the umbilical cord samples (GMT [95% CI] = 98.0 [81.7–116.6]) than in the paired maternal serum (GMT [95% CI] = 81.2 [67.6–97.4]); Figure 3M), with a TR of 113.1% (*p *= 0.0005; Table 3).

### 3.4 Potential Factors Associated With the Reduction in the Placental Transfer of DENV‐Specific Antibodies to the Newborn

We investigated the role of maternal DENV immunity on the transfer of DENV‐specific antibodies across the placenta. The placental TRs of DENV‐specific IgG (TR, 113.3% vs. 120.4%; *p* = 0.01) and DENV‐1 neutralizing antibodies (TR, 82.6% vs. 114.6%; *p *< 0.01) were lower in multiparous mothers compared to nulliparous (Table 4). The same correlation was observed analyzing the parity (Table 4). Interestingly, we found that the placental TRs of DENV‐1 neutralizing antibodies (TR, 114.9% vs. 93.4%; *p *< 0.01) were higher in mother with previously reported DENV infection compared to those without history of DENV (Table 4). The placental TRs (73.9% vs. 114.9%, *p* < 0.01) of DENV‐1 nAbs were lower in vaginal births when compared to C‐section (Table 4).

**Table 4:** Potential maternal factors associated with a decrease in the transplacental transfer of DENV‐specific IgG antibodies and DENV‐1 nAbs to neonates. The characteristics were represented by sample number (*n*) and percentage (%).

Maternal characteristic	No. (%)	IgG anti‐EDIII DENV		No. (%)	nAbs anti‐DENV‐1	
	Total (*n* = 193)	TR^a^, geo mean (95% CI)	*p* value*	Total (*n* = 177)	TR^a^, geo mean (95% CI)	*p* value*
Age (in years)						
14–24	82 (42.5)	113.9 (109.9–118,1)	0.46	79 (44.6)	96.5 (84.1–110.9)	0.60
25–35	92 (47.7)	117.3 (112.5–122.4)		82 (46.3)	91.9 (78.5–107.6)	
> 35	19 (9.8)	114.7 (107.4–121.7)		16 (9.1)	77.1 (52.8–112.6)	
Ethnicity						
White	130 (67.3)	114.1 (110.4–117.8)	0.10	118 (66.7)	90.0 (80.5–100.6)	0.05
Black or mixed	63 (32.6)	118.9 (114.0–124.0)		59 (33.3)	106.0 (90.8–123.8)	
Illness during pregnancy						
GDM						
No	181 (93.8)	115.7 (112.6–118.8)	0.83	166 (93.8)	93.1 (83.9–103.5)	0.37
Yes	12 (6.2)	114.7 (104.5–126.0)		11 (6.2)	82.8 (61.2–111.9)	
HDPs						
No	181 (93.8)	115.9 (112.8–119.0)	0.40	166 (93.8)	94.3 (85.3–104.3)	0.11
Yes	12 (6.2)	111.7 (99.1–125.9)		11 (6.2)	68.5 (39.0–120.6)	
Anemia						
No	181 (93.8)	115.9 (113.0–119.0)	0.97	165 (93.2)	93.1 (84.3–102.9)	0.91
Yes	12 (6.2)	110.8 (95.0–129.2)		12 (6.8)	84.1 (45.0–157.3)	
Syphilis						
No	178 (92.2)	115.8 (112.7–119.0)	0.85	163 (92.1)	93.4 (84.7–103.1)	0.18
Yes	11 (7.8)	114.0 (104.9–123.9)		11 (6.2)	113.4 (63.4–203.1	
Toxoplasmosis						
No	111(57.5)	117.2 (113.3–121.2)	0.28	102 (57.6)	100.0 (87.8–113.9)	0.20
Yes	65 (33.7)	114.4 (109.0–120.1)		60 (33.9)	89.1 (76.0–104.4)	
Gravidity^b^						
Primipara	63 (32.8)	120.4 (115.3–125.9)	0.01	61 (34.5)	114.6 (99.0–132.4)	< 0.01
Multipara	130 (67.2)	113.3 (109.8–117.0)		116 (65.5)	82.6 (72.6–93.9)	
Parity^c^						
Nulliparity	70 (36.3)	121.1 (116.3–126.1)	< 0.01	68 (38.4)	116.5 (101.8–133.4)	< 0.01
Multiparity	123 (63.7)	112.6 (109.0–116.3)		109 (61.6)	80.0 (70.1–91.4)	
Type of birth						
Vaginal	95 (49.2)	114.4 (110.3–118.6)	0.33	87 (50.6)	73.9 (62.7–87.0)	< 0.01
C‐section	98 (50.8)	116.8 (112.6–121.2)		90 (49.4)	114.9 (103.9–127.0)	
History of DENV infection						
*No*	143 (74.1)	115.5 (112.2–118.9)	0.66	132 (74.6)	93.4 (84.6–103.1)	< 0.01
*Yes*	50 (25.9)	116.0 (109.6–122.7)		90 (25.4)	114.9 (103.9–127.0)	
17D YF vaccination^d^						
*No*	114 (59.1)	114.2 (110.6–117.9)	0.14	103 (58.2)	94.8 (82.8–108.5)	0.46
*Yes*	75 (38.9)	118.0 (112.7–123.5)		70 (39.5)	89.7 (76.5–105.1)	

Abbreviations: CI, confidence interval; DENV, dengue virus; DENV‐1, dengue virus serotype 1; EDIII, envelope protein domain III; GDM, gestational diabetes mellitus; HDPs, hypertensive disorders in pregnancy; IgG, immunoglobulin G; nAbs, neutralizing antibodies; TR, transfer rate; YF, yellow fever; %, percentage.

^a^ Transfer rate (TR) = (neonatal nAb titer/maternal nAb titer) × 100.

^b^ Gravidity = total number of pregnancies.

^c^ Parity = the number of full‐term children borne by a woman, excluding miscarriages or abortions in early pregnancy but including stillbirths.

^d^ Missing data for 4 participants.

* *p* values were determined by the nonparametric Mann–Whitney test or Kruskal–Wallis test when appropriate.

Low birthweight babies (< 2500 g) had significantly lower transfer ratios compared to heavier babies (Table 5), for both DENV IgG anti‐EDIII (TR = 103.0 vs. 116.2, *p* = 0.04) and for nAbs against DENV‐1 (TR = 54.5 vs. 94.8, *p* = 0.01). Additionally, a reduction in the TR of DENV‐1 nAbs was noted in infants with a birth length of less than 46 cm (TR = 54.0 vs. 95.0, *p* < 0.01) and in pre‐term newborns (TR = 64.3.5 vs. 93.9, *p* = 0.04, Table 5). Due to the small sample size (*n* = 17 pairs) and the high uniformity of TR in the positive pairs for ZIKV (Table 3), we could not analyze factors potentially associated with reduced placental transfer of ZIKV‐specific antibodies to neonates.

**Table 5:** Potential neonatal factors associated with a decrease in the transplacental transfer of DENV‐specific IgG antibodies and DENV‐1 nAbs to neonates. The characteristics were represented by sample number (*n*) and percentage (%).

Neonatal characteristic	No (%)	IgG anti‐EDIII DENV		No (%)	nAbs anti‐DENV‐1	
	Total (*n* = 193)	TR,^a^ geo mean (95% CI)	*p* value*	Total (*n* = 177)	TR,^a^ geo mean (95% CI)	*p* value*
Weight (g)						
< 2500	8 (4.1)	**99.9 (89.4–111.6)**	**0.02**	8 (4.5)	**54.5 (30.7–96.8)**	**0.01**
≥ 2500	185 (95.9)	**116.2 (113.2–119.3)**		169 (95.5)	**94.8 (85.7–104.9)**	
Length (cm)^b^						
< 46	9 (4.7)	105.7 (95.1–117.5)	0.12	9 (5.1)	**54.0 (32.9–88.5)**	**< 0.01**
≥ 46	183 (94.8)	116.2 (113.1–119.3)		167 (94.4)	**95.0 (85.9–105.3)**	
Gestational age (week)						
< 37 (preterm)	11 (5.7)	109.9 (102.0–118.3)	0.29	11 (6.2)	**64.3 (44.1–93.8)**	**0.04**
≥ 37 (term)	182 (94.3)	116.0 (112.9–119.1)		166 (93.8)	**93.9 (84.7–104.21)**	
Sex						
Male	89 (46.1)	114.5 (110.4–118.9)	0.60	81 (45.8)	90.2 (76.8–106.0)	0.70
Female	104 (53.9)	116.5 (112.4–120.8)		96 (54.2)	94.4 (83.1–107.1)	
Apgar indices (1 min)						
0–6	6 (3.1)	115.1 (101.2–131.0)	0.95	6 (3.4)	70.7 (38.5–129.9)	0.15
7–10	187 (96.9)	115.7 (112.6–118.7)		171 (96.6)	93.4 (84.3–103.4)	
GA × birthweight						
SGA	13 (6.7)	106.3 (98.2–115.2)	0.21	11 (6.2)	88.2 (51.2–151.8)	0.34
AGA	156 (80.8)	116.2 (112.9–119.6)		141 (79.7)	96.6 (86.7–107.6)	
LGA	24 (12.5)	116.8 (108.3–126.3)		21 (11.9)	76.8 (54.0–109.2)	
Neonatal RDS						
No	165 (85.5)	115.0 (112.0–118.1)	0.43	153 (86.4)	91.7 (82.4–102.1)	0.80
Yes	28 (14.5)	119.1 (108.9–130.2)		24 (15.6)	97.1 (72.5–130.1)	
Dermatological changes						
No	70 (36.3)	115.8 (111.3–120.6)	0.77	67 (37.8)	99.0 (84.6–115.8)	0.40
Yes	123 (63.7)	115.5 (111.7–119.4)		110 (62.1)	88.7 (77.9–101.0)	

Abbreviations: CI, confidence interval; DENV, dengue virus; DENV‐1, dengue virus serotype 1; EDIII, envelope protein domain III; GA, gestational age; IgG, immunoglobulin G; nAbs, neutralizing antibodies; RDS, respiratory distress syndrome; TR, transfer rate; %, percentage.

^a^ Transfer rate (TR) = (neonatal nAb titer/maternal nAb titer) × 100.

b Lack of information on the length and head circumference of 4, 2 newborns, respectively.

* *p* values were determined by the nonparametric Mann–Whitney test or Kruskal–Wallis test when appropriate.

## 4 Discussion

Arboviruses are emerging and re‐emerging infectious diseases endemic/epidemic in various regions worldwide, including Brazil [1]. The high potential for arbovirus spread and its impact on public health are highlighted by recent epidemics of Zika virus (ZIKV), dengue virus (DENV), and chikungunya virus (CHIKV) in the Americas [11]. Currently, the epidemiological burden and the public health impact of ZIKV and DENV in Brazil remain inadequately understood in the general population and among high‐risk subpopulations, such as pregnant women [12]. The risks associated with ZIKV and DENV infections during pregnancy are relevant due to the potential vertical transmission and fetal complications [13]. Moreover, there are few studies investigating the transplacental transfer of anti‐ZIKV and anti‐DENV antibodies from Brazilian mothers to their newborns.

This cross‐sectional study conducted in São Paulo city provided seroprevalence rates for ZIKV and DENV among parturients as of January 2020. Specifically, the seroprevalence for ZIKV was found to be 2.8% (95% CI: 1.8%–4.5%), while that for DENV was 10‐fold higher at 31.6% (95% CI: 28.0%–35.4%). The low ZIKV seroprevalence observed in this study contrasts sharply with higher rates reported in the Northeast of Brazil, with 63% in pregnant women in Salvador (2015–2016) [14] and 30% among Bolivian blood donors (2016–2017) [15]. However, our findings align with lower rates reported in Southeast Brazil, including 7.2% in postpartum women in Santos (2016–2017) [16] and 5.6%–9.1% among blood donors in Ribeirão Preto (2016–2017) [10]. Regional factors, such as São Paulo's notably low ZIKV incidence (0.5 cases per 100 000 in 2016 vs. the national average of 63.9) [17], prior DENV immunity [18], socioeconomic vulnerabilities [19], and vector adaptation [20], may further explain the uneven distribution of ZIKV circulation. In addition, particular attention should be paid to technological aspects when comparing flavivirus seroprevalence studies, as discrepancies may result from methodological differences, as IgG detection by ELISA may overestimate prevalence due to cross‐reactivity with other flaviviruses [21].

The limited literature on DENV seroprevalence in the São Paulo region constrains comparisons with our results, as available studies are sparse, geographically dispersed, and largely outdated [22]. Importantly, Northeastern Brazilian cities, like Recife and Salvador consistently reported high DENV prevalence of over 90% [14, 23]. Globally, a 2000–2019 meta‐analysis estimated an average DENV seroprevalence of 38%, with regional rates of 53% in the Americas, consistent with Brazil's 40%, though rates vary by subpopulations (e.g., 27% in pregnant women, 33% in general population and 52% in blood donors) [12].

The low seroprevalence of ZIKV and DENV in this study underlines both the relative protection the region has benefited to date for climatic and entomological reasons, and the vulnerability of the São Paulo population to these infections. The rapid proliferation of *Aedes aegypti* and *Aedes albopictus* [24], driven by climate change, urbanization, and insecticide resistance, exacerbates the arboviral risk [25, 26]. São Paulo's dense urban population and prevalence of heat islands further contribute to arbovirus outbreaks [27], as evidenced by the recent outbreaks of DENV [28] and YFV [29], underscoring the need for robust surveillance and control measures. Mathematical models indicate that young women of reproductive age are at higher risk during future ZIKV outbreaks [30], presenting a significant public health challenge.

ZIKV and DENV seropositivity were significantly associated with maternal birthplace, marital status, educational attainment, and infection history. Mothers from Brazil's Northeast region, which remains hyperendemic for DENV and was the epicenter of the 2015–2016 Zika outbreak [31], exhibited higher seropositive rates than those from the South and Southeast regions. This disparity reflects the Northeast's historically high arbovirus prevalence and migratory patterns to São Paulo since the 1930s. Women in stable relationships had higher ZIKV and DENV seroprevalence, potentially due to sexual transmission of ZIKV [32] and low condom use. Additionally, there may be an increase likelihood of intrafamily and intradomicile DENV transmission. Lower educational attainment was also linked to increased DENV seropositivity, aligning with studies associating limited education with higher infection risk due to reduced awareness of prevention and etiology [33], but also linked to socioeconomic level and the limited access to protective devices. Infection history correlated strongly with seropositivity, underscoring the immune response and test accuracy. However, many seropositive participants reported no history of infection, which is consistent with the known frequency of asymptomatic cases and reinforces the value of seroepidemiological studies over traditional surveillance systems.

No clinical characteristics of the newborns were linked to maternal ZIKV seropositivity, likely due to the absence of recent infections during pregnancy. The lack of correlation between ZIKV seropositive mothers and newborns with microcephaly may be due to several factors, as ZIKV is not the only cause of this condition. Maternal nutrition and genetic factors, may potentially affect fetal brain development and increase the risk of microcephaly [34]. In addition to ZIKV, other infections such as rubella, cytomegalovirus (CMV), and toxoplasmosis, as well as exposure to toxins, medications, or alcohol during pregnancy, can also lead to microcephaly [35, 36]. For DENV seropositivity, a correlation was observed only with dermatological abnormalities. Although specific skin changes linked to maternal IgG are not consistently documented, a nonhaemolytic mechanism could be involved, since some maternal antibodies, may interfere with neonatal liver enzymes or bilirubin metabolism, exacerbating dermatological abnormalities, as jaundice.

The efficient placental transfer of ZIKV‐ and DENV‐specific maternal IgG to the fetus observed in the study population aligns with findings from other studies [37, 38, 39]. These results underscore the active transport of IgG across the placenta, a well‐characterized immunological mechanism mediated by FcRn receptors [40]. Similarly, the efficient placental transfer of nAbs against ZIKV and DENV‐3 serotype was confirmed. Our investigation into the influence of maternal DENV immunity on the placental transfer of DENV‐specific antibodies revealed significant patterns shaped by factors such as maternal parity, prior DENV exposure, and mode of delivery. Low birthweight, small size and preterm babies showed reduced TR. Low transfer of antibodies has been previously described in LBW [41] and preterm babies [42] and is probably due to an immature placenta lacking Fcg II receptors. Mothers infected a long‐time ago, and older mothers would be more likely to not transmit dengue antibodies, as they would have lower antibody levels. This would reduce the risk of DHF in their infants.

This study has some limitations. The primary limitation is its unicenter design, conducted at a single hospital in western São Paulo, which may affect the accuracy of seroprevalence data and limit generalizability to all pregnant women or the broader city population. Efforts to include more hospitals were hindered by logistical challenges related to participant enrollment, sample collection, and clinical data gathering. Additionally, the small number of ZIKV‐positive parturients precluded analysis of chance effects on variables and identification of factors associated with reduced transplacental transport to newborns. This limitation was an unforeseen consequence of the findings. Nevertheless, the sample size was representative of the study population, supporting data validity. Furthermore, serological IgM anti‐ZIKV and anti‐DENV antibodies were only assessed in maternal sera with nAbs titres (≥ 40), potentially underestimating true IgM prevalence, since it was assumed that IgM antibodies have a high neutralizing potential [43]. Lastly, anti‐YFV antibodies could not be assessed in ZIKV‐ and DENV‐positive samples, limiting the exclusion of cross‐reactivity. However, cross‐reactivity with YFV is likely minimal due to the absence of reported prior YFV infections, widespread YFV vaccination among participants, and data showing no correlation between YFV vaccination and ZIKV/DENV seropositivity. Recent studies also indicate low YFV cross‐reactivity compared to ZIKV and DENV [44, 45].

## Author Contributions

**Rafael Rahal Guaragna Machado:** conceptualization, methodology, formal analysis, investigation, data curation, writing – original draft, writing – review and editing, visualization. **Danielle Bruna Leal Oliveira:** conceptualization, writing – review and editing. **Silvia Maria Ibidi:** conceptualization, resources, writing – review and editing. **Edison Luiz Durigon:** conceptualization, resources, writing – review and editing, supervision, funding acquisition. **Danielle Bastos Araujo:** methodology, investigation, writing – review and editing. **Marcella Sanches Peres:** investigation, writing – review and editing. **Samuel Santos Pereira:** investigation, writing – review and editing. **Ralyria Mello:** investigation, writing – review and editing. **Camila Soares Pereira:** investigation, writing – review and editing. **Boris Pastorino:** investigation, writing – review and editing. **Michele da Silva Jordan Faleiros:** investigation, resources, writing – review and editing. **Rossana Pulcineli Vieira Franscisco:** resources, writing – review and editing. **Carlos Tadashi Yoshizaki:** resources, writing – review and editing. **Xavier de Lamballerie:** resources, writing – review and editing. **Luís Carlos de Sousa Ferreira:** resources, writing – review and editing, funding acquisition.

## Ethics Statement

Ethical approval was obtained from the Ethics Committees in Research with Human Beings of the University Hospital of the University of São Paulo (CEPSH/HU‐USP—CAAE: 79966617.2.3001.0076; approval no 2.971.333) and from the Institute of Biomedical Sciences of the University of São Paulo (CEPSH/ICB—CAAE: 79966617.2.0000.5467; approval no 2.633.620; CAAE: 66796223.0.0000.5467; approval no 5.897.747). Written informed consent was obtained from all participants or their legal guardians (in the case of minors). All the samples collected in this study were stored in a biorepository (CEPSH‐ICB approval no 029.2022).

## Conflicts of Interest

The authors declare no conflicts of interest.

## Supporting information

Supplemental data.

## Data Availability

The data that support the findings of this study are available on request from the corresponding author. The data are not publicly available due to privacy or ethical restrictions.
