# Supplementary material for: Seroprevalence and Placental Transfer of Zika and Dengue Virus Antibodies in Postpartum Women in Southeast Brazil
Source: J Med Virol. 2025 May 21;97(5):e70384. doi: 10.1002/jmv.70384 (PMC12096061; doi:10.1002/jmv.70384)

**Supplemental data**

**Figure S1.** Geographical distribution of the 601 participants' residences within the São Paulo metropolitan region (RMSP). The RMSP is constituted by the following cities: São Paulo, Osasco, Santo André, Taboão da Serra, Cotia, Barueri, Santana de Parnaíba, Cajamar, Ferraz de Vasconcelos, Carapicuíba and Itapevi. São Paulo city is subdivided into six demographic regions (North, South, East, West, Central, and Southeast), within which HU-USP is situated in the West region of the city The neighbourhoods of Butantã, Rio Pequeno, Morumbi, Jaguaré, Raposo Tavares and Vila Sônia (outlined in red), which are part of the western region of the city of São Paulo, are shown in detail, as this area contains the majority of the participants' homes. The red cross symbol indicates the location of the University Hospital of the University of São Paulo (HU-USP). The image was created using the QGIS 3.6.1 software.


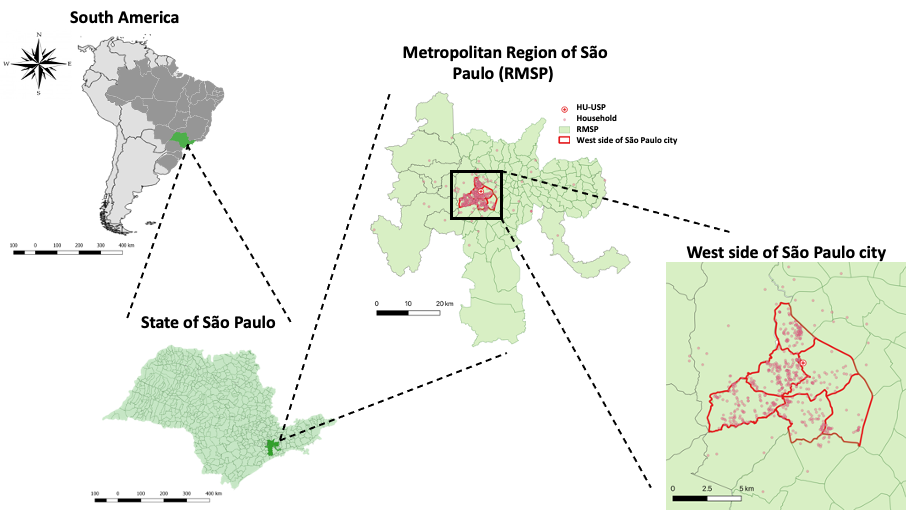


# Figure S2. Flowchart showing application of study inclusion and exclusion criteria.

**
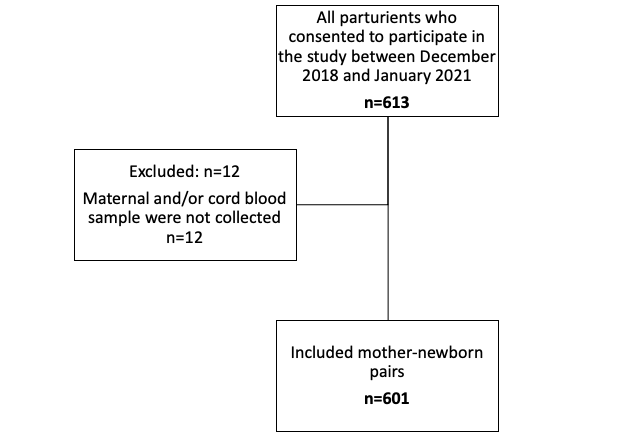
**

**Table S1.** Potential risk factors for ZIKV and DENV seropositive mothers. Univariable and multivariable regression analysis of place of birth, marital status and education level of the 601 parturients participating in the study potentially correlated with their seropositivity to ZIKV (n=17) or DENV (n=193). The characteristics were represented by sample number (n) and percentage (%).

| **Maternal characteristic** | **ZIKV** | | | | | | **DENV** | | | | | |
| --- | --- | --- | --- | --- | --- | --- | --- | --- | --- | --- | --- | --- |
|  | **Seropositive (n=17)** | **Seronegative (n=584)** | **Univariable regression*** | | **Multivariable regression#** | | **Seropositive (n=190)** | **Seronegative (n=411)** | **Univariable regression*** | | **Multivariable regression#** | |
|  |  |  | **OR (95% CI)** | ***p* value** | **OR (95% CI)** | ***p* value** |  |  | **OR (95% CI)** | ***p* value** | **OR (95% CI)** | ***p* value** |
| **Place of birth** |  | | | | | | | | | | | |
| Brazil | 16 (94.1) | 575 (98.5) | - | - | - | - | 189 (99.5) | 402 (97.8) | - | - | - | - |
| *Northeast region* | 13 (81.3) | 143 (24.9) | Ref | - | Ref | - | 78 (41.3) | 78 (19.4) | Ref | - | Ref | - |
| *South or Southeast region* | 3 (18.7) | 422 (73.4) | **12.79 (3.59-45.52)** | **<0.01** | **14.72 (4.31-70.69)** | **<0.01** | 105 (55.5) | 320 (79.6) | **3.05 (2.08-4.47)** | **<0.01** | **2.86 (1.94-4.23)** | **<0.01** |
| *North or Midwest region* | 0 (0.0) | 10 (1.7) | 1.97 (0.11-35.60) | 0.64 | 1.40 (0.18-30.61) | 0.78 | 6 (3.2) | 4 (1.0) | 0.67 (0.18-2.45) | 0.54 | **4.45 (1.23-17.87)** | **0.02** |
| **Marital status** |  | | | | | | | | | | | |
| Living together (consensual union) | 16 (94.1) | 400 (68.5) | Ref | - | Ref | - | 143 (75.3) | 273 (66.4) | Ref | - | Ref | - |
| Not living together | 1 (5.9) | 184 (31.5) | 7.36 (0.97-55.92) | 0.05 | 6.82 (1.16-132.7) | 0.08 | 47 (24.7) | 138 (33.6) | **1.54 (1.04-2.27)** | **0.02** | 1.35 (0.90-2.05) | 0.15 |
| **Education level, years** |  | | | | | | | | | | | |
| <9 (NS, IES, CES and/or IMS) | 1 (5.9) | 106 (18.1) | Ref | - | Ref | - | 43 (22.6) | 64 (15.6) | Ref | - | Ref | - |
| ≥9 e <12 (CMS and/or IHS) | 4 (23.5) | 157 (26.9) | 0.37 (0.04-3.36) | 0.38 | 0.37 (0.02-2.83) | 0.39 | 55 (28.9) | 106 (25.8) | 1.29 (0.78-2.15) | 0.32 | 1.35 (0.89-2.05) | 0.16 |
| ≥12 (CHS, IC and/or CC) | 12 (70.6) | 321 (55.0) | 0.25 (0.03-1.96) | 0.19 | 0.23 (0.01-1.47) | 0.19 | 92 (48.4) | 241 (58.6) | **1.76 (1.12-2.77)** | **0.01** | **1.78 (1.10-2.89)** | **0.01** |

Abbreviations: nAbs, neutralizing antibodies; IQR, Interquartile range; NS, No schooling; IPS, Incomplete elementary school; CES, Complete elementary school; IMS, Incomplete middle school; CMS, Complete middle school; IHS, Incomplete high school; CHS, Completed high school; IC, Incomplete college; CC, Complete college; CI, Confidence interval; OR, Odds ratio; Ref, reference; ZIKV, Zika virus; DENV, Dengue virus.

**p* values ​​were calculated using Fisher's exact test (two-sided method).

# The multiple logistic regression analysis was conducted using a main-effects model with an intercept. The log-likelihood ratio (G^2^) hypothesis test was statistically significant, leading to the rejection of the null hypothesis (p<0.0001).

**Table S2.** Pregnancy and obstetric characteristics of the participants (n=601). Potential risk factors for parturients with and without anti-ZIKV or anti-DENV nAbs detected.

| **Maternal characteristic** | **No. (%)** | **ZIKV** | | | | **DENV** | | | |
| --- | --- | --- | --- | --- | --- | --- | --- | --- | --- |
|  | **Total (n=601)** | **Seropositive (n=17)** | **Seronegative (n=584)** | **OR (95% CI)** | ***p* value*** | **Seropositive (n=190)** | **Seronegative (n=411)** | **OR (95% CI)** | ***p* value*** |
| **Use of legal and/or illegal drugs** | | | | | | | | | |
| No | 589 (98.0) | 17 (100.0) | 572 (97.9) | Ref | - | 186 (97.9) | 403 (98.1) | Ref | - |
| Yes | 12 (2.0) | 0 (0.0) | 12 (2.1) | 0.00 (0.00-10.13) | >0.99 | 4 (2.1) | 8 (1.9) | 1.08 (0.36-3.78) | >0.99 |
| **Illness during pregnancy** | | | | | | | | | |
| **Flu (common cold)** |  | | | | | | | | |
| No | 414 (68.9) | 9 (52.9) | 405 (69.3) | Ref | - | 133 (70.0) | 281 (68.4) | Ref | - |
| Yes | 187 (31.1) | 8 (47.1) | 179 (30.7) | 2.01 (0.78-4.92) | 0.18 | 57 (30.0) | 130 (31.6) | 0.93 (0.64-1.34) | 0.70 |
| **Gestational diabetes mellitus (GDM)** |  | | | | | | | | |
| No | 553 (92.0) | 17 (100.0) | 536 (91.8) | Ref | - | 178 (93.7) | 375 (91.2) | Ref | - |
| Yes | 48 (8.0) | 0 (0.0) | 48 (8.2) | 0.00 (0.00-2.47) | 0.38 | 12 (6.3) | 36 (8.8) | 0.70 (0.36-1.39) | 0.33 |
| **Hypertensive disorders in pregnancy (HDPs)** | | | | | | | | | |
| No | 565 (94.0) | 16 (94.1) | 549 (94.0) | Ref | - | 178 (93.7) | 387 (94.2) | Ref | - |
| Yes | 36 (6.0) | 1 (5.9) | 35 (6.0) | 0.98 (0.09-6.13) | >0.99 | 12 (6.3) | 24 (5.8) | 1.09 (0.55-2.15) | 0.85 |
| **Urinary tract infection (UTI)** |  | | | | | | | | |
| No | 414 (68.9) | 10 (58.8) | 404 (69.2) | Ref | - | 126 (66.3) | 288 (70.1) | Ref | - |
| Yes | 187 (31.1) | 7 (41.2) | 180 (30.8) | 1.57 (0.60-4.30) | 0.42 | 64 (33.7) | 123 (29.9) | 1.19 (0.83-1.71) | 0.39 |
| **Anemia** |  | | | | | | | | |
| No | 571 (95.0) | 16 (94.1) | 555 (95.0) | Ref | - | 178 (93.7) | 393 (95.6) | Ref | - |
| Yes | 30 (5.0) | 1 (5.9) | 29 (5.0) | 1.19 (0.11-7.64) | 0.58 | 12 (6.3) | 18 (4.4) | 1.47 (0.70-3.01) | 0.31 |
| **Human papillomavirus (HPV)** |  | | | | | | | | |
| No | 596 (99.2) | 17 (100.0) | 579 (99.1) | Ref | - | 188 (98.9) | 408 (99.3) | Ref | - |
| Yes | 05 (0.8) | 0 (0.0) | 5 (0.9) | 0.00 (0.00-25.15) | >0.99 | 2 (1.1) | 3 (0.7) | 1.45 (0.25-7.13) | 0.65 |
| **Syphilis** |  | | | | | | | | |
| No | 575 (95.7) | 17 (100.0) | 558 (95.5) | Ref | - | 182 (95.8) | 393 (95.6) | Ref | - |
| Yes | 26 (4.3) | 0 (0.0) | 26 (4.5) | 0.00 (0.00-4.98) | >0.99 | 8 (4.2) | 18 (4.4) | 0.96 (0.43-2.24) | >0.99 |
| **Gestation** | | | | | | | | | |
| Primipara | 231 (38.6) | 12 (70.6) | 219 (37.6) | Ref | - | 64 (33.7) | 167 (40.6) | Ref | - |
| Multipara | 370 (61.4) | 5 (29.4) | 365 (62.4) | **0.25 (0.10-0.70)** | **<0.01** | 126 (66.3) | 244 (59.4) | 1.35 (0.94-1.94) | 0.10 |
| **Parity** | | | | | | | | | |
| Nulliparity | 252 (41.9) | 12 (70.6) | 240 (41.1) | Ref | - | 72 (37.9) | 180 (43.8) | Ref | - |
| Multiparity | 349 (58.1) | 5 (29.4) | 344 (58.9) | **0.29 (0.11-0.81)** | **0.02** | 118 (62.1) | 231 (56.2) | 1.28 (0.90-1.80) | 0.18 |
| **Type of birth** | | | | | | | | | |
| Vaginal | 305 (50.7) | 10 (58.8) | 295 (50.5) | Ref | - | 96 (50.5) | 209 (50.8) | Ref | - |
| C-section | 296 (49.3) | 7 (41.2) | 289 (49.5) | 0.71 (0.27-1.95) | 0.62 | 94 (49.5) | 202 (49.2) | 1.01 (0.72-1.42) | >0.99 |
| **Prenatal care visits †** | | | | | | | | | |
| Minimum (>5) | 557 (92.7) | 15 (88.2) | 542 (92.8) | Ref | - | 176 (92.6) | 381 (92.7) | Ref | - |
| Inadequate (<4) | 37 (6.1) | 2 (11.8) | 35 (6.0) | 2.06 (0.45-8.15) | 0.28 | 14 (7.4) | 23 (5.6) | 1.32 (0.66-2.56) | 0.46 |

Abbreviations: nAbs, neutralizing antibodies; CI, Confidence interval; OR, Odds ratio; Ref, reference; ZIKV, Zika virus; DENV, Dengue virus

* The p-values were calculated using Fisher's exact test (two-sided method). OR Confidence intervals (CIs) were computed using the Baptista-Pike method. †Missing data of seven participants.

**Table S3.** Clinical characteristics of the parturients (n=601) potentially correlated with ZIKV seropositivity (n=17) and DENV seropositivity (n=190).

| **Maternal characteristic** | **No (%)** | **ZIKV** | | | | | | **DENV** | | | | | |
| --- | --- | --- | --- | --- | --- | --- | --- | --- | --- | --- | --- | --- | --- |
|  | **Total (n=601)** | **Soropositive (n=17)** | **Soronegative (n=584)** | **Univariable regression** | | **Multivariable regression** | | **Soropositive (n=190)** | **Soronegative (n=411)** | **Univariable regression** | | **Multivariable regression** | |
|  |  |  |  | **OR (95% CI)** | ***p* value** | **OR (95% CI)** | ***p* value** |  |  | **OR (95% CI)** | ***p value*** | **OR (95% CI)** | ***P value*** |
| **Symptoms for arboviral diseases** | | | | | | | | | | | |  |  |
| Rash | | | | | | | | | | | | | |
| *No* | 587 (97.7) | 17 (100) | 570 (97.6) | Ref | - | - | - | 186 (97.9) | 401 (97.6) | Ref | - | - | - |
| *Yes* | 14 (2.3) | 0 (0.0) | 14 (2.4) | 0.89 (0.05-15.52) | >0.99 | - | - | 4 (2.1) | 10 (2.4) | 0.86 (0.29-2.56) | >0.99 | - | - |
| Fever | | | | | | | | | | | | | |
| *No* | 549 (91.4) | 16 (94.1) | 533 (91.3) | Ref | - | - | - | 176 (92.4) | 373 (90.7) | Ref | - | - | - |
| *Yes* | 52 (8.6) | 1 (5.9) | 51 (8.7) | 0.65 (0.06-3.97) | >0.99 | - | - | 14 (7.4) | 38 (9.3) | 0.78 (0.42-1.45) | 0.53 | - | - |
| Arthralgia | | | | | | | | | | | | | |
| *No* | 542 (90.2) | 14 (82.3) | 528 (90.4) | Ref | - | - | - | 173 (91.0) | 369(89.8) | Ref | - | - | - |
| *Yes* | 59 (9.8) | 3 (17.7) | 56 (9.6) | 2.02 (0.60-7.06) | 0.22 | - | - | 17 (9.0) | 42 (10.2) | 0.86 (0.49-1.55) | 0.66 | - | - |
| Myalgia | | | | | | | | | | | | | |
| *No* | 499 (83.0) | 14 (82.3) | 485 (83.1) | Ref | - | - | - | 162 (85.3) | 337 (82.0) | Ref | - | - | - |
| *Yes* | 102 (17.0) | 3 (17.7) | 99 (16.9) | 1.05 (0.31-3.57) | >0.99 | - | - | 28 (14.7) | 74 (18.0) | 0.78 (0.48-1.25) | 0.35 | - | - |
| Headache | | | | | | | | | | | | | |
| *No* | 324 (53.9) | 13 (70.6) | 312 (53.4) | Ref | - | Ref | - | 105 (55.3) | 219 (53.3) | Ref | - | - | - |
| *Yes* | 277 (46.1) | 5 (29.4) | 272 (46.6) | 0.44 (0.17-1.80) | 0.15 | 0.45 (0.14-1.24) | 0.14 | 85 (44.7) | 192 (46.7) | 0.92 (0.65-1.30) | 0.66 | - | - |
| Conjunctival hyperemia | | | | | | | | | | | | | |
| *No* | 570 (94.8) | 15 (88.2) | 555 (95.0) | Ref | - | - | - | 181 (95.3) | 389 (94.6) | Ref | - | - | - |
| *Yes* | 31 (5.2) | 2 (11.8) | 29 (5.0) | 2.55 (0.55-10.28) | 0.21 | - | - | 9 (4.7) | 22 (5.4) | 0.88 (0.39-1.97) | 0.84 | - | - |
| Lymphadenopathy | | | | | | | | | | | | | |
| *No* | 510 (84.9) | 15 (88.2) | 495 (84.8) | Ref | - | - | - | 169 (88.9) | 341 (83.0) | Ref | - | Ref | - |
| *Yes* | 91 (15.1) | 2 (11.8) | 89 (15.2) | 0.74 (0.17-2.77) | >0.99 | - | - | 21 (11.1) | 70 (17.0) | 0.60 (0.36-1.01) | 0.06 | 0.57 (0.31-1.02) | 0.06 |
| **Exposure during pregnancy** | | | | | | | | | | | | | |
| Travel | | | | | | | | | | | | | |
| *No* | 344 (57.2) | 10 (58.8) | 334 (57.2) | Ref | - | - | - | 124 (65.3) | 220 (53.5) | Ref | - | Ref | - |
| *Yes* | 257 (42.8) | 7 (41.2) | 250 (42.8) | 0.93 (0.36-2.55) | >0.99 | - | - | 66 (34.7) | 191 (46.5) | **0.61 (0.43-0.87)** | **<0.01** | **0.61 (0.41-0.90)** | **0.01** |
| Mosquito bite | | | | | | | | | | | | | |
| *No* | 305 (50.8) | 11 (64.7) | 294 (50.3) | Ref | - | - | - | 98 (51.6) | 207 (50.4) | Ref | - | - | - |
| *Yes* | 296 (49.2) | 6 (35.3) | 290 (49.7) | 0.55 (0.21-1.50) | 0.32 | - | - | 92 (48.4) | 204 (49.6) | 0.95 (0.68-1.34) | 0.79 | - | - |
| Use of repellent | | | | | | | | | | | | | |
| *No* | 302 (50.3) | 12 (70.6) | 290 (49.7) | Ref | - | Ref | - | 103 (54.2) | 199 (48.4) | Ref | - | Ref | - |
| *Yes* | 299 (49.7) | 5 (29.4) | 294 (50.3) | 0.41 (0.16-1.14) | 0.13 | 0.45 (0.14-1.25) | 0.14 | 87 (45.8) | 212 (51.6) | 0.79 (0.56-1.11) | 0.18 | 0.87 (0.60-1.28) | 0.49 |
| **History of infection** | | | | | | | | | | | | | |
| DENV | | | | | | | | | | | | | |
| *No* | 541 (90.0) | 15 (88.2) | 526 (90.1) | Ref | - | - | - | 141 (74.2) | 400 (97.3) | Ref | - | Ref | - |
| *Yes* | 60 (10.0) | 2 (11.8) | 58 (9.9) | 1.21 (0.27-5.42) | 0.80 | - | - | 49 (25.8) | 11 (2.7) | **12.6 (6.5-24.6)** | **<0.01** | **13.74 (7.1-29.0)** | **<0.01** |
| ZIKV | | | | | | | | | | | | | |
| *No* | 600 (99.8) | 16 (94.1) | 584 (100) | Ref | - | - | - | 190 (100) | 410 (99.8) | Ref | - | - | - |
| *Yes* | 1 (0.2) | 1 (5.9) | 0 (0.0) | **106.3 (4.2-2707.7)** | **<0.01** | - | - | 0 (0.0) | 1 (0.2) | 0.72 (0.03-17.71) | 0.84 | - | - |
| **Vaccination^#^** | | | | | | | | | | | | | |
| Yellow fever | | | | | | | | | | | | | |
| *No* | 348 (57.9) | 11 (64.7) | 337 (56.8) | Ref | - | - | - | 116 (60.4) | 232 (55,4) | Ref | - | - | - |
| *Yes* | 253 (42.1) | 6 (35.3) | 247 (43.2) | 0.74 (0.27-2.02) | 0.62 | - | - | 74 (39.6) | 179 (44.6) | 0.83 (0.58-1.17) | 0.28 | - | - |

**Table S4.** Correlation of prenatal maternal serology for human immunodeficiency virus (HIV), syphilis, hepatitis B (HBV), toxoplasmosis, and group B streptococcus (GBS) with seropositivity for ZIKV (n=17) and DENV (n=190). The data are represented as sample number (n) and percentage (%).

| **Prenatal maternal serology** | **Total n (%)** | **ZIKV** | | | | **DENV** | | | | | |
| --- | --- | --- | --- | --- | --- | --- | --- | --- | --- | --- | --- |
|  |  | **Soropositive (n=17)** | **Soronegative (n=584)** | **Univariable regression*** | | **Soropositive (n=190)** | **Soronegative (n=411)** | **Univariable regression*** | | **Multivariable regression#** | |
|  |  |  |  | **OR (95% CI)** | ***p* value** |  |  | **OR (95% CI)** | ***p* value** | **OR (95% CI)** | ***p* value** |
| **HIV** | 601 (100) |  | | | | | | | | | |
| Negative | 601 (100) | 17 (100) | 584 (100) | N/A | - | 190 (100) | 411 (100) | N/A | - | - | - |
| Positive | 0 (0.0) | 0 (0.0) | 0 (0.0) | N/A | - | 0 (0.0) | 0 (0.0) | N/A | - | - | - |
| **Syphilis** | 591 (98.3) |  | | | | | | | | | |
| Negative | 565 (95.6) | 14 (82.3) | 551 (94.3) | Ref | - | 175 (92.1) | 390 (94.9) | Ref | - | Ref | - |
| Positive | 26 (4.4) | 1 (5.9) | 25 (4.3) | 1.57 (0.14-9.00) | 0.49 | 11 (5.8) | 15 (3.6) | 1.63 (0.73-3.73) | 0.27 | 1.63 (0.71-3.61) | 0.23 |
| **Hepatitis B** | 566 (94.2) |  | | | | | | | | | |
| Negative | 559 (98.8) | 13 (76.5) | 546 (93.5) | Ref | - | 171 (90.0) | 388 (94.4) | Ref | - | - | - |
| Positive | 7 (1.2) | 0 (0.0) | 7 (1.2) | 0.00 (0.00-27.38) | >0.99 | 2 (1.0) | 5 (1.2) | 0.91 (0.17-4.25) | >0.99 | - | - |
| **Toxoplasmosis** | 544 (90.5) |  | | | | | | | | | |
| Negative | 378 (69.5) | 10 (58.8) | 368 (63.0) | Ref | - | 110 (57.9) | 268 (65.2) | Ref | - | Ref | - |
| Positive | 166 (30.5) | 5 (29.4) | 161 (27.6) | 1.14 (0.43-3.10) | 0.78 | 63 (33.1) | 103 (25.1) | **1.49 (1.01-2.18)** | **0.04** | **1.48 (1.01-2.18)** | **0.04** |
| **GBS** | 309 (51.4) |  | | | | | | | | | |
| Negative | 223 (72.2) | 5 (29.4) | 218 (37.3) | Ref | - | 69 (36.3) | 154 (37.5) | Ref | - | - | - |
| Positive | 86 (27.8) | 2 (11.8) | 84 (14.4) | 1.04 (0.20-4.95) | >0.99 | 25 (13.1) | 61 (14.8) | 0.91 (0.53-1.59) | 0.78 | - | - |

Abbreviations: HIV, human immunodeficiency virus; GBS, group B streptococcus; ZIKV, Zika virus; DENV, Dengue virus.

***The p-values were determined by Fisher's Exact Test.

#The multiple logistic regression analysis was conducted using a main-effects model with an intercept. The log-likelihood ratio (G^2^) hypothesis test was statistically significant, leading to the rejection of the null hypothesis (p<0.0001).

**Table S5.** Potential risk factors for newborns from DENV seropositive mothers. Univariable and multivariable regression analysis of apgar score, dermatological changes, neonatal screening test of the 609 newborns participating in the research potentially correlated with seropositivity to DENV (n=193). The clinical characteristics were represented by sample number (n) and percentage (%).

| **Neonatal characteristic** | **No (%)** | **DENV** | | | | | |
| --- | --- | --- | --- | --- | --- | --- | --- |
|  | **Total (n=609)** | **Seropositive (n=193)** | **Seronegative (n=416)** | **Univariable regression*** | | **Multivariable regression#** | |
|  |  |  |  | **OR (95% CI)** | ***p* value** | **OR (95% CI)** | ***p* value** |
| **Apgar indices (1 min)** | | | | | | | |
| 7-10 | 583 (95.7) | 187 (96.9) | 388 (95.1) | Ref | - | Ref | - |
| 4-6 | 16 (2.6) | 2 (1.0) | 14 (3.4) | 4.67 (0.66-32.74) | 0.12 | 0.33 (0.05-1.20) | 0.14 |
| 0-3 | 10 (1.6) | 4 (2.1) | 6 (1.5) | 1.38 (0.38-4.96) | 0.62 | 1.43 (0.36-5.12) | 0.58 |
| **Dermatological changes** | | | | | | | |
| No | 275 (45.2) | 70 (36.3) | 206 (49.5) | Ref | - | Ref | - |
| Yes | 334 (54.8) | 123 (63.7) | 210 (50.5) | **1.72 (1.22-2.43)** | **<0.01** | **1.65 (1.16-2.35)** | **<0.01** |
| **Hip dysplasia (Ortolani test)** | | | | | | | |
| Negative | 593 (97.4) | 192 (95.5) | 401 (96.4) | Ref | - | Ref | - |
| Positive | 16 (2.6) | 1 (0.5) | 15 (3.6) | 0.14 (0.02-1.06) | 0.05 | 0.14 (0.00-0.73) | 0.06 |
| **Neonatal screening** | | | | | | | |
| Red reflex test (RRT) | | | | | | | |
| *Normal* | 605 (99.3) | 3 (1.5) | 1 (0.2) | Ref | - | Ref | - |
| *Abnormal* | 4 (0.7) | 190 (98.5) | 411 (99.8) | 6.49 (0.67-62.80) | 0.11 | 5.05 (0.64-102.7) | 0.16 |

Abbreviations: OR, odds ratio; CI, confidence interval; %, percentage; Ref, reference; ZIKV, Zika virus; DENV, Dengue virus.

**p* values ​​were calculated using Fisher's exact test (two-sided method).

# The multiple logistic regression analysis was conducted using a main-effects model with an intercept. The log-likelihood ratio (G^2^) hypothesis test was statistically significant, leading to the rejection of the null hypothesis (p<0.0001).

**Table S6**. Performance of the Zika-v IgG kit and the in-house anti-DENV EDIII IgG ELISA compared to VNT_100_ in the 601 serum samples analyzed. Sensitivity specificity positive and negative predictive values likelihood ratio and AUC were calculated. Values are presented as whole numbers and/or percentages (%), and the 95% confidence interval (95% CI) was calculated.

| **Parameters** | **∆NS1-based ZIKV IgG ELISA** | | **EDIII-based DENV IgG ELISA** | |
| --- | --- | --- | --- | --- |
|  | **VNT_100_** | | **VNT_100_** | |
|  | Positive (titer ≥ 40) | Negative (titer < 40) | Positive (titer ≥ 40) | Negative (titer < 40) |
| Positive (OD ≥ 0.23 or 0.30)* | 12 | 179 | 179 | 18 |
| Negative (OD < 0.23 or 0.30)* | 05 | 11 | 11 | 393 |
| Sensitivity [95%CI] | 76.5% (13/17) [52.7%-90.4%] | | 94.2% (179/190) [89.9%-96.7%] | |
| Specificity [95%CI] | 98.3% (574/584) [96.9%-99.1%] | | 95.6% (393/411) [93.2%-97.2%] | |
| Predictive positive value [95%CI] | 56.5% (13/23) [36.8%-74.4%] | | 90.9% (179/197) [86.0%-94.1%] | |
| Predictive negative value [95%CI] | 99.3% (574/578) [98.2%-99.7%] | | 97.3% (393/404) [95.2%-98.5%] | |
| Likelihood ratio (LR) | 44.7 | | 21.5 | |
| AUC ± SE [95%CI] | 0.90 ± 0.01 [0.89-0.95] | | 0.97 ± 0.01 [0.95-0.98] | |

* Based on the reactivity values obtained and using the test cut-off of 0.23 (anti-ZIKV ΔNS1 IgG) and 0.30 (anti-DENV 1-4 EDIII IgG), calculated from the ROC curve, the number of positive and negative samples was determined.

Abbreviations: OD, optical density; CI, confidence interval; AUC, area under the curve; SE, standard error; NS1, non-structural protein 1; EDIII, envelope domain III; VNT100, antibody titer capable of neutralizing 100% of the cytopathic effect caused by the virus; ZIKV, Zika virus; DENV, Dengue virus.

**Figure S3.** ROC curve and scatter plot of anti-ZIKV NS1 IgG and anti-DENV EDIII IgG serum levels compared to VNT100 results. **(A and B)** ROC curve demonstrating the performance of **(A)** the ∆NS1-based ZIKV IgG ELISA test and **(B)** the in-house EDIII-based DENV IgG ELISA test. The reference line is represented in red (---). **(C and D)** Scatter plot of absorbance values (OD 450 nm) obtained from samples testing positive (VNT100 ≥ 40) or negative (VNT100 < 40) for the presence of nAbs **(C)** anti-ZIKV or **(D)** anti-DENV. **(E and F)** Comparison of absorbance values greater than or equal to, or lower than the cutoff, and the VNT results for **(E)** ZIKV and **(F)** DENV. The black dotted horizontal line indicates the cutoff value (0.23 for ZIKV and 0.30 for DENV). The p-value was determined using the non-parametric Mann-Whitney test. OD, optical density; AUC, area under the curve; ns, not significant; *, p<0.05; **, p<0.01; ***, p<0.001; ****, p<0.0001.


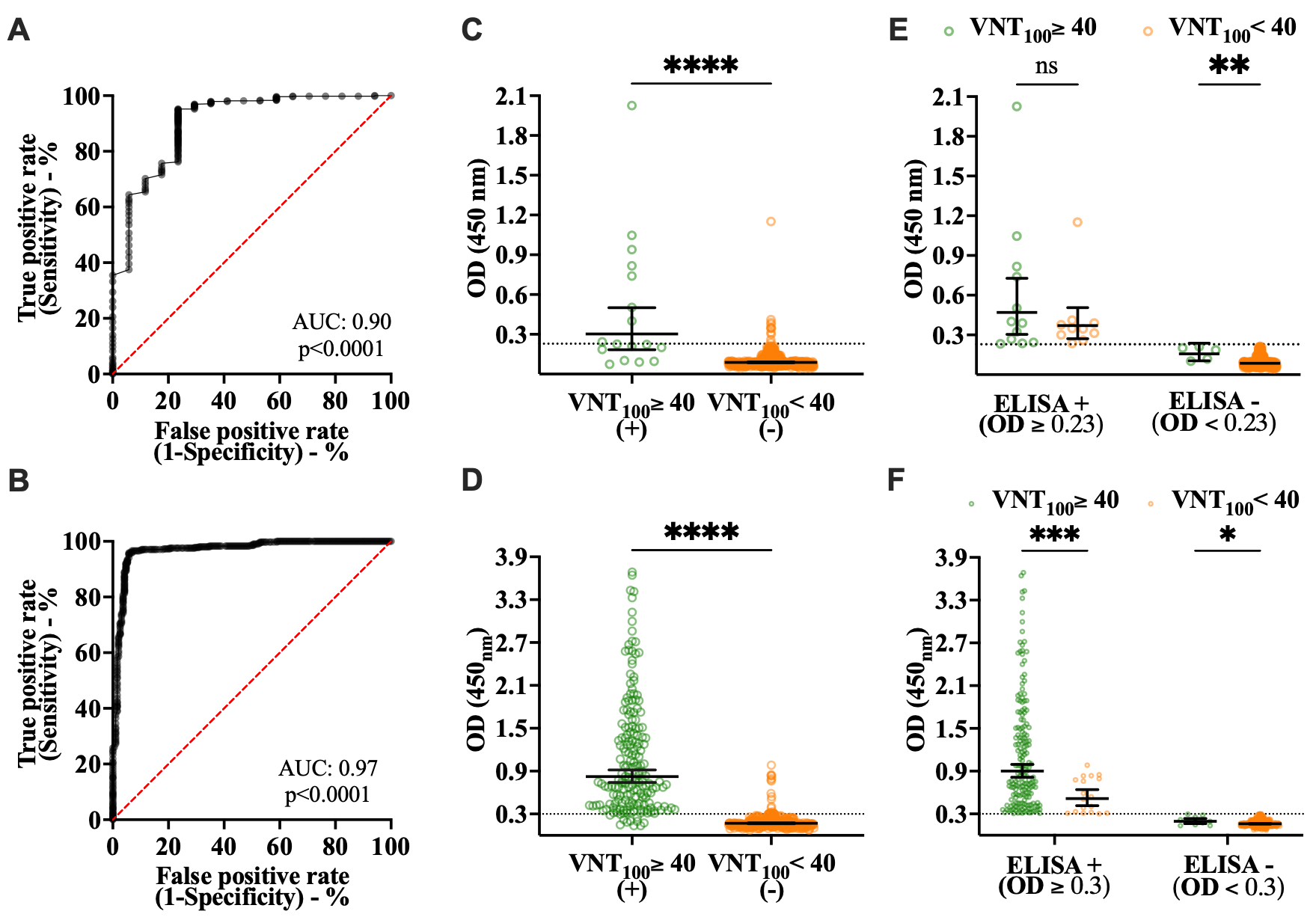

Supplement: Supplementary file 1 — Supplemental data. [file JMV-97-e70384-s001.docx]
